# Lay-Up and Consolidation of a Composite Pipe by In Situ Ultrasonic Welding of a Thermoplastic Matrix Composite Tape

**DOI:** 10.3390/ma11050786

**Published:** 2018-05-11

**Authors:** Riccardo Dell’Anna, Francesca Lionetto, Francesco Montagna, Alfonso Maffezzoli

**Affiliations:** Department of Engineering for Innovation, University of Salento, via per Monteroni, Lecce 73100, Italy; riccardo.dellanna@unisalento.it (R.D.A.); francesco.montagna@unisalento.it (F.M.); alfonso.maffezzoli@unisalento.it (A.M.)

**Keywords:** thermoplastic composites, filament winding, ultrasonic welding, polyethylene terephthalate, thermoplastic composite pipe

## Abstract

In this work, the potential of preformed thermoplastic matrix composite tapes for the manufacturing of composite pipes by filament winding assisted by in situ ultrasonic welding was evaluated. Unidirectional tapes of E-glass-reinforcedamorphous poly (ethylene terephthalate) were laid up and consolidated in a filament winding machine that was modified with a set-up enabling ultrasonic welding. The obtained composite specimens were characterized by means of morphological and dynamic mechanical analysis as well as void content evaluation, in order to correlate welding parameters to composite properties.

## 1. Introduction

The interest in continuous fiber-reinforced thermoplastic composites is continuously increasing due to their inherent benefits compared with thermoset composites such as damage tolerance, high impact resistance, chemical and solvent resistance, unlimited shelf life, low storage costs, weldability, and recyclability [1,2,3]. Other key advantages of thermoplastic composites include the potential of fast in situ out-of-autoclave processing, by applying pressure and heat in one step, and the potential for automation with significant decrease of work time and costs [4,5]. For these reasons, fiber-reinforced thermoplastic composites are continuously replacing traditional materials like metals and wood in many applications [6,7]. On the other hand, one of the main problem of thermoplastic composites is the high viscosity of the molten matrix, which leads to a difficult impregnation of the fiber bundle. Many manufacturing processes for thermoplastic composites have been developed to solve the problem, and the use of a prepreg is commonly preferred in industrial practice [8].

A recent and promising application of thermoplastic matrix composites is in pipelines, especially in the offshore industry, where high corrosion resistance, high stiffness-to-weight ratio, and low maintenance costs are crucial. Moreover, the high strains to failure of thermoplastic matrices make thermoplastic composite pipes more flexible than traditional thermosetting composite pipes and suitable for deep-water applications [9,10]. Typical matrices used in the production of thermoplastic composite pipes are PolyEthylene (PE), PolyPropylene (PP), PolyAmide (PA), PolyVinylidene DiFluoride (PVDF), and PolyEther Ether Ketone (PEEK) [11,12,13]. To the authors’ knowledge, there are no studies on the use of reinforced Poly (Ethylene Terephthalate) (PET) for pipelines, although its excellent mechanical and thermal properties, good chemical resistance, and barrier properties make PET a very suitable matrix for manufacturing composite pipes.

Composite pipes are usually produced by filament winding, which, in the case of thermoplastic matrix composites, requires a well-designed modification in order to lay up and consolidate the plies usually by means of fusion bonding which involves the application of heat and pressure [14,15,16]. The heat source used for melting the thermoplastic matrix plays a key role in the efficiency of the process, the final quality of the parts, and the processing costs [14,17]. Among the bonding techniques used for thermoplastic matrix composites, ultrasonic welding is very attractive for composite processing and joining, since it is characterized by a low-energy input, highly localized heating, very short welding times, and ease of automation [18,19,20]. During ultrasonic welding, the parts to be joined are held together under pressure and subjected to ultrasonic vibrations in the 20–40 kHz range [21,22]. The heat is produced at the joint interface through a combination of surface and intermolecular friction which leads to matrix melting. The use of ultrasound for automated fiber placement has been recently proposed as an alternative to hot gas torch, laser, and infrared heating [23]. Moreover, ultrasonic wave propagation has the potential for online process monitoring [24,25].

The authors have recently proposed an ultrasonic assisted filament winding technology for commingled E-glass/Polypropylene rovings [26]. In the present work, a different matrix and a different preform, consisting in unidirectional reinforced tape, has been studied. According to the literature, the use of preformed tape should reduce the consolidation times caused by the high viscosities of thermoplastic melts [27]. The aim of the present work is to evaluate the potential of preformed thermoplastic matrix composite tapes for the manufacturing of composite pipes by filament winding assisted by in situ ultrasonic welding. The idea underlying the proposed technology is to overcome some critical issues associated to in situ consolidation, such as the temperature control, considering the high temperature needed for matrix melting, and fiber deposition on curved surfaces. The proposed technology can also find application in composite repair and piping joining. Unidirectional tapes of E-glass reinforced amorphous poly (ethylene terephthalate), named LPET, have been laid up and consolidated in a filament winding machine modified with a set-up enabling ultrasonic welding. A preformed tape, instead of the commingled roving used in the previous work, has been used. The composite pipes have been characterized by morphological and dynamic mechanical analysis and void content evaluation in order to find the optimum welding parameters.

## 2. Materials and Methods

### 2.1. Equipment for Lay-Up and Ultrasonic Welding

The developed set-up for continuous ultrasonic welding of thermoplastic tape was a filament winding machine, produced by VEM SpA (Italy), with two degrees of freedom: the rotation of the metal mandrel and the translation of the carriage. The machine was modified with the integration of an ultrasonic horn and a steel roller for compacting the overlapping layers. The titanium ultrasonic horn was fixed on the steel frame of the filament winding machine. The horn was connected with the power generator, model ELMD 20 produced by SONIC Italia (Italy), characterized by a maximum power of 2000 W and a work frequency of 20 kHz. The contact force between the horn and welding partners was controlled by compressed air.

**Figure 1:**
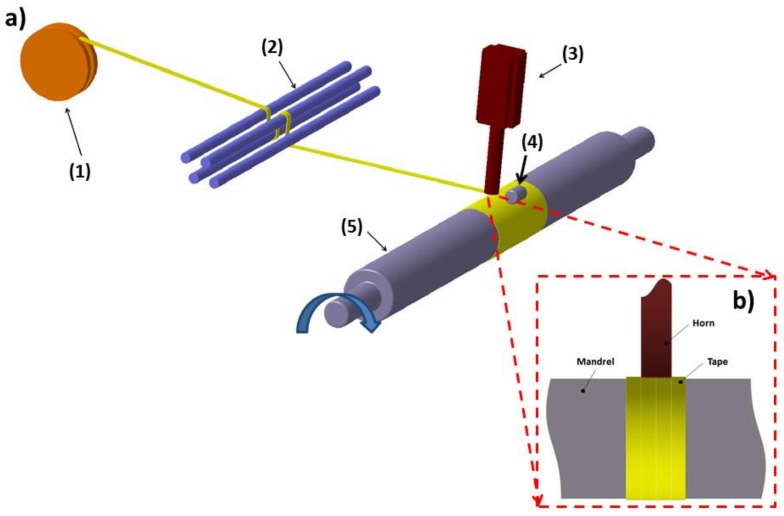
(**a**) Schematic representation (not in scale) of the experimental set-up for ultrasonic assisted consolidation with spool (1), tensioning system (2), ultrasonic horn (3), compaction roller (4), mandrel (5); (**b**) front view of the horn in contact with the tape wound on the mandrel.

As reported in Figure 1a, the E-glass/LPET tape, unwound from the spool (1), went through the tensioning system (2). At the nip point on the mandrel, the tape was in contact with the ultrasonic horn (3), which simultaneously applied pressure and ultrasonic energy. After the ultrasonic heating, welding was completed through a compaction roller (4) which applied further pressure to the tape. Hoop winding was set (with an angle of 87.6°) in order to produce a pipe with fibers oriented in the hoop direction. The sonotrode diameter (40 mm) was larger than the tape width (19 mm), allowing the side welding of each tape with the adjacenttape, as reported in Figure 1b. Different mandrel speeds, ranging between 0.13 rad/s and 0.52 rad/s, were used to consolidate the composite pipe.

### 2.2. Materials

The material used was a pre-consolidated unidirectional tape, produced by COMFIL Aps (Denmark), made of E-glass fibers and amorphous poly (ethylene terephthalate), LPET, with a fiber volume content of 42%. The tape had a nominal width of 19 mm and a thickness of 0.8 mm. The thermal properties of the thermoplastic matrix, not indicated in the technical datasheet, were determined by differential scanning calorimetry using an 822e DSC (Mettler-Toledo SpA, Novate Milanese, Italy) calorimeter through a dynamic scan from 25 °C to 280 °C at 1 °C/min. The thermal degradation of LPET was analyzed by thermogravimetric analysis in air, using a TGA 1 (Mettler-Toledo SpA, Novate Milanese, Italy), from 25 °C to 800 °C at 10 °C/min.

**Figure 2:**
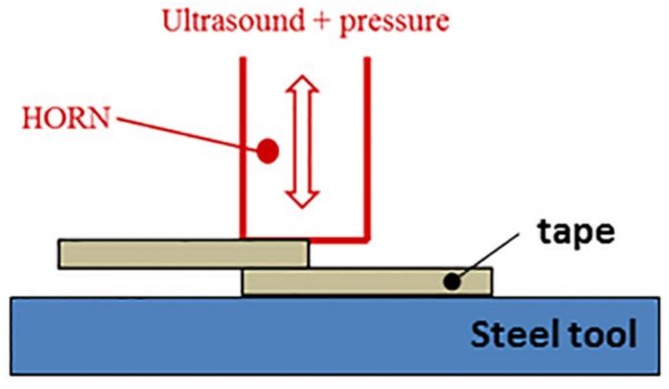
Sketch of ultrasonic spot welding.

Preliminary to pipe consolidation, ultrasonic spot welding was performed to obtain ultrasonic welded and consolidated specimens in a single lap shear configuration according to ASTM D1002 [28]. The scheme of ultrasonic spot welding is shown in Figure 2. Single lap shear tests were performed with the aim of determining the effect of welding time and pressure. A Lloyd 5 KN (Lloyd Instruments Ltd., Bognor Regis, UK) was used with a crosshead speed of 1.3 mm/min.

### 2.3. Characterization

The composite pipes produced by ultrasonic-assisted filament winding at different mandrel speeds were characterized by density measurements, dynamic mechanical analysis and morphological analysis. The density of specimens taken from consolidated pipes was experimentally determined according to the ASTM D792 standard [29]. For each kind of sample, the obtained value was the average of ten specimens with dimensions of 30 × 20 mm^2^. The density of unconsolidated tape was also measured as a reference value. The theoretical density was calculated by the rule of mixtures assuming a density of glass fibers and LPET equal to 2.54 g/cm^3^ [30] and 1.38 g/cm^3^ [31], respectively.

The void content, φ, was obtained by Equation (1) according to the ASTM D2734-03 Standard [32]:

(1) $$\phi =\frac{\rho_{\mathrm{ct}}-\rho_{\mathrm{cr}}}{\rho_{\mathrm{ct}}}\times 100$$

where ρ_ct_ and ρ_cr_ were the theoretical and measured density of the composite specimens, respectively.

Dynamic Mechanical Analysis (DMA) was used to measure the storage and loss component of complex shear modulus G_12_* on specimens taken from consolidated pipes. The specimens were tested in torsion mode on an ARES rheometer (TA Instruments, New Castle, DE, USA). For each pipe consolidated at a different mandrel speed, five samples were cut at 90° with respect to the fiber direction, with dimensions equal to 30 × 10 × 1.6 mm^3^. Specimens of unconsolidated tape were also measured as a reference value. Specimens were tested at 1 Hz upon heating from 30 °C to 250 °C at a heating rate of 2 °C/min under a shear strain of 1%. The use of samples cut at 90° to the fiber direction, done so in order to measure a matrix dominated property (G_12_), allowed to better highlight the effect of processing conditions on welding and consolidation. The limited specimen curvature of samples cut at 90° was negligible, considering that the thickness of the specimens (0.8 mm) was much lower than the mandrel radius, 75 mm. Samples cut parallel to fibers would result strongly curved, and their properties would be less affected by the fabrication process.

Small samples cut from consolidated E-glass/LPET specimens and as received tape were observed by a ZEISS EVO 40 (Carl Zeiss AG, Oberkochen, Germany), Scanning Electron Microscope (SEM), operating with an accelerating voltage of 20 kV at variable air pressure in the measuring chamber.

## 3. Results and Discussion

**Figure 3:**
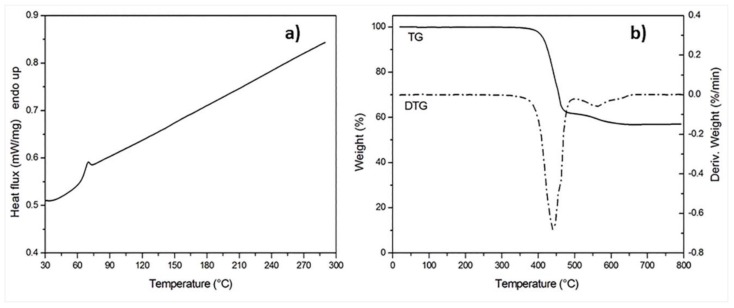
(**a**) DSC and (**b**) TGA thermograms of E-glass/LPET tape during heating at 10 °C/min.

Differential Scanning Calorimetry (DSC) was carried out to evaluate the amorphous behavior of LPET matrix. The DSC thermogram reported in Figure 3a only showed the discontinuity in the baseline associated to the glass transition, which indicates the completely amorphous nature of E-glass/LPET tape. A glass transition temperature of 68 °C was determined by the inflection point method.

ThermoGravimetric Analysis (TGA) was carried out on specimens of E-glass/LPET tape in order to evaluate the maximum processing temperature of LPET matrix. As reported in Figure 3b, there was no weight change in the sample until the temperature reached 350 °C. The temperature of initial degradation, defined as the temperature at which a weight loss of 5% was measured, was found to be 413 °C.

**Figure 4:**
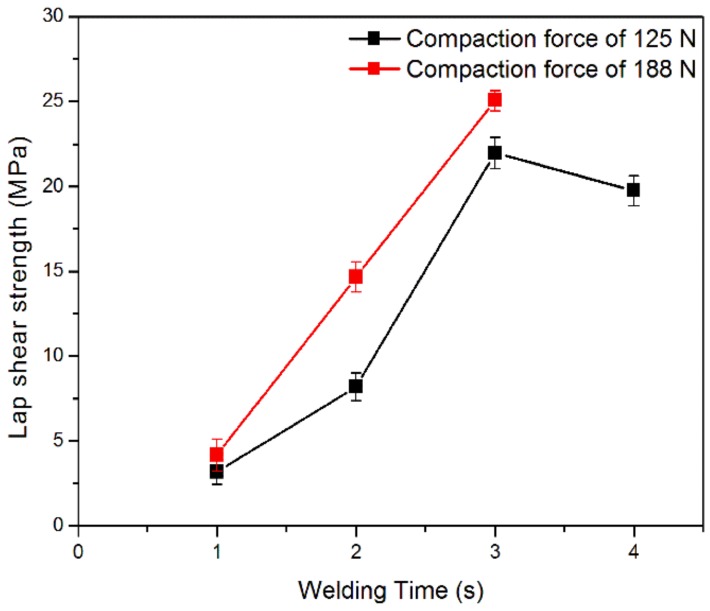
Effect of welding time and force on lap shear strength on ultrasonic spot welded E-glass/LPET specimens.

An ultrasonic spot welding on flat specimens of E-glass/LPET tape was carried out, with the aim of identifying the range of process parameters to use for the production of composite pipe by ultrasonic-assisted filament winding. First of all, the proper horn force was determined through lap shear tests on specimens welded at two different welding forces, 125 N and 188 N, and different welding times, between 1 s and 4 s. The obtained Lap Shear Strength (LSS) values are reported in Figure 4. The increase of the welding force lead to an increase in LSS values, which was more significant for welding times higher than 1 s. Both compaction forces of 125 N and 188 N showed an initial increase of lap shear strength as a function of the welding time, with the maximum values at 3 s. For the specimens welded at 125 N and higher welding time (4 s), resin squeezing was responsible for a decrease in strength.

The LSS values obtained on ultrasonic spot welded E-glass/LPET by using a welding time of 3 s and a force of 188 N, were in the same range (18–25 MPa) as those measured by Warren et al. on resistance welded semi-crystalline PET reinforced with glass fibers [33].

Therefore, a horn compaction force of 188 N and a welding time of 3 s were chosen to maximize the bonding strength between tapes. These data were then exploited to set the proper conditions for a continuous ultrasonic welding and consolidation during filament winding.

The angular speed to be set on the filament winding machine was calculated from the optimized welding time obtained from the ultrasonic spot welding experiments described before:

(2) $$\omega =\frac{l}{r\times t}$$

where *l* was the horn size, *r* the mandrel radius, and *t* the contact time between the welding parameters and the horn.

**Figure 5:**
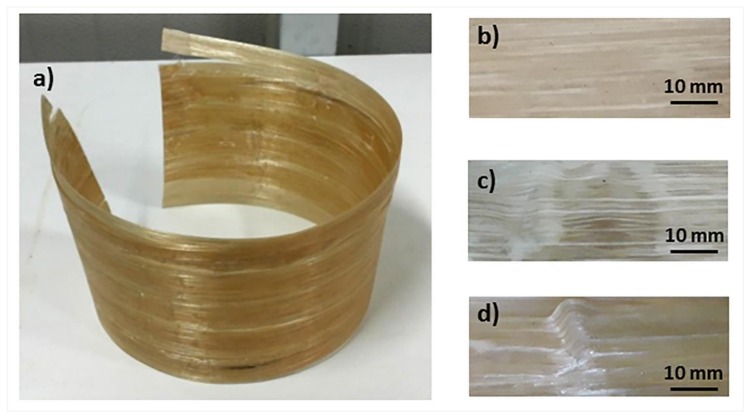
(**a**) Composite cylinder produced by ultrasonic assisted filament winding with mandrel speed of 0.18 rad/s; (**b**) zoom of well consolidated composite part; defects of composites pipes produced with mandrel speed of 0.13 rad/s; (**c**) fiber wrinkling and (**d**) matrix squeezing.

Since the horn size was 40 mm and the mandrel radius was 75 mm, by using a contact time of 3 s, a mandrel speed of 0.18 rad/s was obtained. However, considering the differences between a static and a continuous process, the cylinder prototypes were fabricated by an ultrasonic assisted filament winding with mandrel speeds ranging from 0.13 rad/s to 0.52 rad/s. A cylinder made of two layers is shown in Figure 5a; each one was obtained by welding ten tapes placed side-by-side after the extraction from the mandrel.

In order to evaluate the effect of the mandrel speed on the quality of ultrasonic welding and consolidation, the density was measured and the void content was determined according to Equation (1). The obtained values are reported in Table 1, together with the values obtained on as-received E-glass/LPET tape. The ultrasonic consolidation process was able to lower the initial void content of as-received E-glass/LPET tape when the winding angular speed of the mandrel was 0.18 rad/s, i.e., from 2.02% to 1.76% with a regular and smooth surface and good consolidation quality (Figure 5b). At higher mandrel speeds, a higher void content was observed as a consequence of the limited time available for healing at tapes interface. On the other hand, matrix squeezing or degradation (Figure 5c) was likely to occur at the lowest winding speed (0.13 rad/s), leading to an increase of void content.

**Figure 6:**
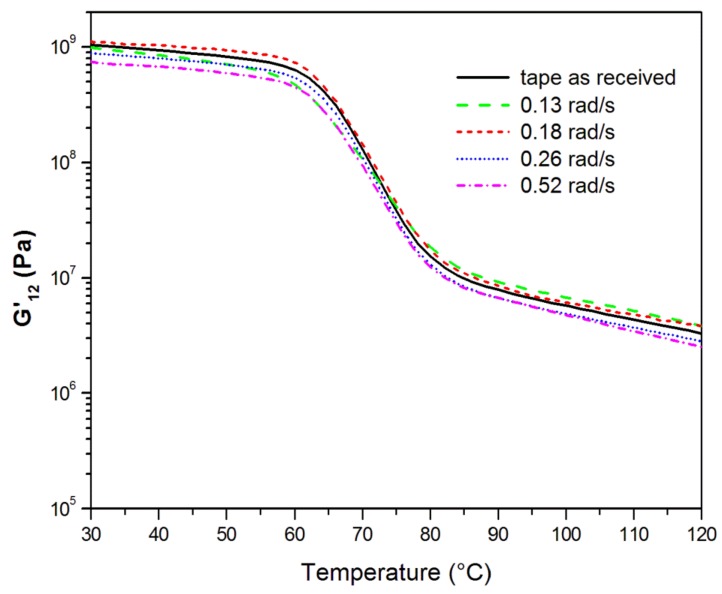
Effect of the temperature on G’_12_ values at 1 Hz for the E-glass/LPET pipes consolidated at different welding speeds.

Dynamic mechanical analysis was used to evaluate the effect of the processing conditions on tape-to-tape consolidation through the measurement of the real and imaginary components of the complex shear modulus G*_12_, a matrix-dominated property. The temperature evolution at 1 Hz of the storage modulus G’_12_, for each ultrasonic welding condition is shown in Figure 6. For comparison purposes, the G’_12_ curve obtained on a specimen of as-received E-glass/LPET tape before the ultrasonic welding is also reported. As the temperature increased during the DMA experiment, G’_12_ decreased for all the composite samples due to the increased molecular mobility of the polymer chains. A drop in the G’_12_ curve was observed in correspondence with the glass transition region between 60 °C and 90 °C. The sample consolidated by ultrasonic welding at a speed of 0.18 rad/s presented the highest storage modulus compared with the other samples, which were consolidated at higher speed. An increase in G’_12_ indicates an improved adhesion between the tapes provided by the ultrasonic welding at the proper mandrel speed.

**Figure 7:**
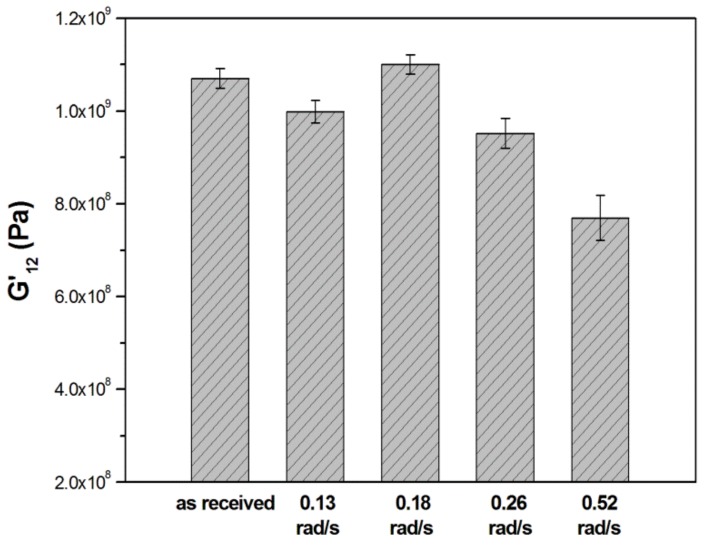
Storage modulus values at 30 °C obtained by DMA at 1 Hz and 2 °C/min.

G’_12_ obtained at 30 °C from the DMA curves are compared in Figure 7. The maximum value, 1.19 GPa, was obtained at a winding speed of 0.18 rad/s. This value is higher than the modulus of the starting E-glass/LPET tape (named “as-received tape” in Table 1), which is produced by a vacuum-assisted consolidation, as reported in the technical datasheet. G’_12_ at 0.13 rad/s was lower than those of unconsolidated tape, probably because at slow mandrel speed, a prolonged contact time between horn and tape can produce a significant fiber wrinkling induced by matrix flow (Figure 5c,d). G’_12_ obtained at 0.26 rad/s was also lower than that of the unconsolidated tape, indicating that this speed did not provide enough time for an efficient consolidation of the E-glass/LPET tapes. By increasing the mandrel speed even more (0.52 rad/s), the bonding between tapes was not properly achieved; this led G’_12_ to be lower than 0.8 GPa, as a consequence of the limited time available for healing at tapes interface. The DMA results confirmed those obtained by the density measurements.

In order to gain further insight into the effect of processing conditions on the shear modulus, the experimental values were compared with the theoretical ones based on the Halpin-Tsai equation and the inverse rule of mixture [34], as reported in Table 2. The highest experimental value was closer to that obtained when applying the inverse rule of mixture, which underestimated the shear modulus. This indicated that the void content, although quite low (Table 1), was able to reduce the shear modulus below the expected value.

**Figure 8:**
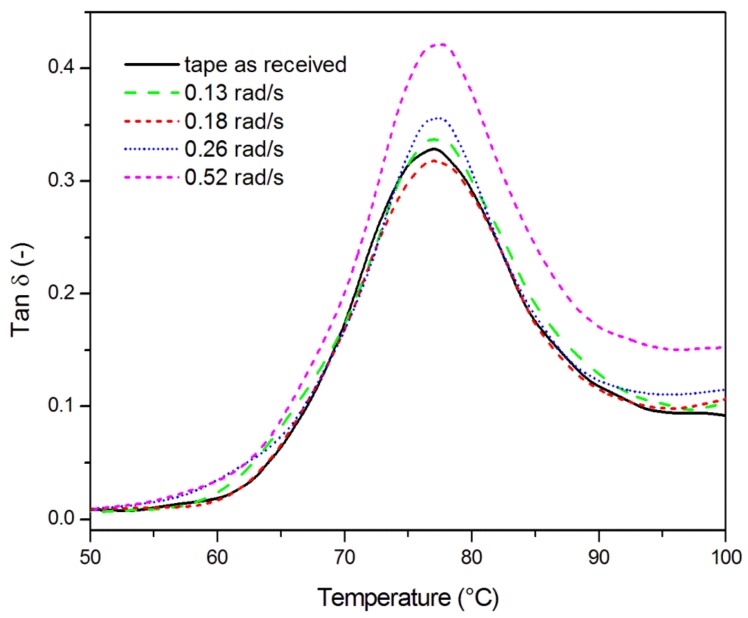
Effect of the temperature on tan δ values at 1 Hz for the E-glass/LPET pipes consolidated at different welding speeds.

A dynamic mechanical analysis enabled the evaluation of tape-to-tape adhesion through the analysis of tan δ curves. The effect of the temperature on tan δ curves at 1 Hz for the E-glass/LPET pipes consolidated at different welding speeds is shown in Figure 8. The peak observed during a DMA experiment at 1 Hz and 2 °C/min, centered around 77 °C, was associated to the glass transition. This was also confirmed by the DSC analysis reported in Figure 1. It is known from literature that an increased height of tan δ peak indicates an increased energy dissipation at the fiber-matrix interface, which can be associated to a poor interfacial adhesion. Moreover, the broadening of the tan δ peak can be an indication of a more heterogeneous structure [35]. As observed in Figure 8, the height of the tan δ peak of the specimen welded at 0.18 rad/s is lower than the peak height of the as-received tape. Moreover, the height and width of the tan δ peak at 0.18 rad/s were the lowest among the specimens welded at different mandrel speeds. Therefore, the loss factor curves were a further demonstration that the speed of 0.18 rad/s was able to provide a good consolidation, with mechanical properties being comparable or even superior to those of the starting preformed tape.

**Figure 9:**
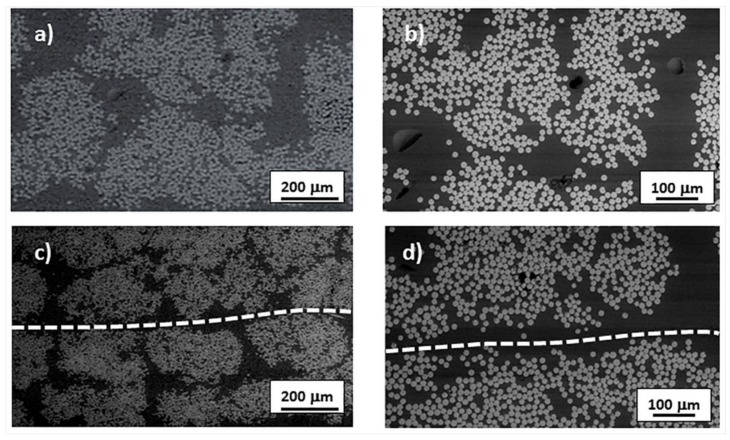
(**a**,**b**) Cross section of unconsolidated E-glass/LPET tape as received; (**c**,**d**) cross section of two E-glass/LPET tapes after ultrasonic assisted filament winding at 0.18 rad/s.

The morphology of the composite samples consolidated at 0.18 rad/s and analyzed by Scanning Electron Microscopy (SEM), was compared in Figure 9 with that of as received tape. The cross section of unconsolidated E-glass/LPET tape (Figure 9a,b) showed fibers not uniformly distributed in the specimen, leading to resin-rich and fiber-rich areas. The porosity, present even in the starting E-glass/LPET tape, which was produced by vacuum technology, was mainly in the form of macro voids located in polymer rich regions.

There was no great difference in the SEM morphology of the specimens consolidated at 0.18 rad/s, where the welding line, indicated by the dashed white line, was observable. The number of macro voids was lower in the consolidated sample, confirming the lover void content determined by density measurements.

## 4. Conclusions

An experimental set-up, integrating a laboratory filament winding machine with an ultrasonic welder, was used to lay up and consolidate a composite pipe by in situ ultrasonic welding of a preformed thermoplastic matrix composite tape. During the winding, a thermoplastic tape was simultaneously in contact with the mandrel and the horn. The horn was able to melt the matrix locally and to apply a pressure on the consolidating material.

The obtained physical, mechanical, and microstructural results confirm the reliability of the proposed technology for the in situ consolidation of thermoplastic semi-preg during filament winding.

The choice of a glass-reinforced tape allowed a good consolidation, both due to perfect matching of the tape during the winding and an excellent propagation of ultrasonic waves during the consolidation.

## 5. Patents

The equipment for lay-up and ultrasonic welding has been patented “Method for the production of composite materials and its applications for the manufacture of products and components in composite materials”, F. Lionetto, F. Montagna, A. Maffezzoli, IT patent number 1420575, 2016.

## Figures and Tables

**Table 1:** Density and void content of E-glass/LPET pipes consolidated by ultrasonic welding at different mandrel speeds, compared with the values of the as-received starting tape.

Winding Speed (rad/s)	Density (g/cm^3^)	Void Content (%)
**0.13**	1.818 ± 7.801 × 10^−3^	2.555 ± 4.190 × 10^−3^
**0.18**	1.832 ± 8.876 × 10^−3^	1.756 ± 4.748 × 10^−3^
**0.26**	1.824 ± 8.029 × 10^−3^	2.233 ± 4.521 × 10^−3^
**0.52**	1.807 ± 4.081 × 10^−3^	3.144 ± 2.190 × 10^−3^
**As received tape**	1.828 ± 5.076 × 10^−3^	2.019 ± 2.312 × 10^−3^

**Table 2:** Theoretical shear modulus of unidirectional E-glass/LPET tape.

Theoretical or Experimental Origin	G’_12_ (GPa)
Inverse rule of mixtures	1.03
Halpin-Tsai equation	1.42
Exp. measurement at 0.18 rad/s	1.19
